# Salt‐dependent regulation of archaellins in *Haloarcula marismortui*

**DOI:** 10.1002/mbo3.718

**Published:** 2018-10-01

**Authors:** Alexey S. Syutkin, Marleen van Wolferen, Alexey K. Surin, Sonja‐Verena Albers, Mikhail G. Pyatibratov, Oleg V. Fedorov, Tessa E. F. Quax

**Affiliations:** ^1^ Institute of Protein Research Russian Academy of Sciences Pushchino Russia; ^2^ Molecular Biology of Archaea Faculty of Biology University of Freiburg Freiburg Germany

**Keywords:** archaea, archaeal flagellum, archaellum, ecoparalogs, halophile, motility

## Abstract

Microorganisms require a motility structure to move towards optimal growth conditions. The motility structure from archaea, the archaellum, is fundamentally different from its bacterial counterpart, the flagellum, and is assembled in a similar fashion as type IV pili. The archaellum filament consists of thousands of copies of N‐terminally processed archaellin proteins. Several archaea, such as the euryarchaeon *Haloarcula marismortui*, encode multiple archaellins. Two archaellins of *H. marismortui* display differential stability under various ionic strengths. This suggests that these proteins behave as ecoparalogs and perform the same function under different environmental conditions. Here, we explored this intriguing system to study the differential regulation of these ecoparalogous archaellins by monitoring their transcription, translation, and assembly into filaments. The salt concentration of the growth medium induced differential expression of the two archaellins. In addition, this analysis indicated that archaellation in *H. marismortui* is majorly regulated on the level of secretion, by a still unknown mechanism. These findings indicate that in archaea, multiple encoded archaellins are not completely redundant, but in fact can display subtle functional differences, which enable cells to cope with varying environmental conditions.

## 1 INTRODUCTION

Motility is one of the key features of living organisms, enabling them to move in the direction of the most favorable growth conditions. Archaea swim with the help of a rotating filament, the archaellum (formerly “archaeal flagellum”). The archaellum is functionally similar to the bacterial flagellum, but their protein composition and structural organization are completely different (Jarrell & Albers, 2012). The archaellum is assembled in a similar fashion as type IV pili (Albers & Jarrell, 2015). As archaea thrive in a high variety of habitats, ranging from extreme, such as high temperature or high salt, to mesophilic environments, such as the deep sea oceans and the human gut, they are major players in diverse ecosystems (DeLong & Pace, 2001; Gaci, Borrel, Tottey, O'Toole, & Brugère, 2014; Hugon, Lagier, Colson, Bittar, & Raoult, 2017; Leininger et al., 2006). Unfortunately, knowledge on regulation of archaellation is incomplete and limited to a few model archaea.

In the crenarchaea *Sulfolobus solfataricus* and *S. acidocaldarius*, it was shown that archaellation is induced by starvation, that is, the growth in absence of tryptone (Lassak et al., 2012; Szabó et al., 2007). In further work on *S. acidocaldarius*, a complex network of both positive and negative regulators of archaellation was revealed (Lassak, Peeters, Wróbel, & Albers, 2013; Reimann et al., 2012). These regulatory proteins are only conserved in organisms of the phylum *Sulfolobales*.

Studies of methanogenic euryarchaea have shown that archaellum biosynthesis is induced at low hydrogen pressure, which is an electron donor in methanogenesis, the main source of energy for these organisms (Hendrickson et al., 2008; Mukhopadhyay, Johnson, & Wolfe, 2000). Changes in temperatue influence archaellum expression in the archaeon *Methanococcus maripaludis* (Ding et al., 2016). In addition, the first transcriptional regulator of archaellation in *Euryarchaea* was identified in this organism (Ding et al., 2016). As for another large group of euryarchaeota, the halophiles, the regulation of archaellum biosynthesis, is not fully understood. The only information about regulation of archaellation in halophiles comes from the model *Haloferax volcanii*. It was shown that there is post‐translational regulation of archaellation, because the presence of the conserved H‐domain of pilins is required for correct archaellum assembly (Esquivel & Pohlschröder, 2014).

In contrast to the regulation of archaellation, the structure and biochemical composition of the archaellum have lately been mapped in increasingly greater detail (Banerjee, Neiner, Tripp, & Albers, 2013; Banerjee et al., 2015; Briegel et al., 2017; Chaudhury et al., 2016; Daum et al., 2017; Poweleit et al., 2016; Reindl et al., 2013). The protein components of the archaellum are quite conserved amongst motile archaea. However, there are some distinct differences between the archaellum operons of archaea belonging to the major phyla *Euryarchaea* and *Crenarchaea*. Euryarchaea generally encode multiple archaellins, the constituents of the archaellum filament (Albers & Jarrell, 2015; Pohlschröder, Ghosh, Tripepi, & Albers, 2011). These are made as preproteins and cleaved by a type III peptidase (Albers, Szabó, & Driessen, 2003; Bardy & Jarrell, 2003; Tripepi, Imam, & Pohlschröder, 2010). This raises the question why euryarchaea need several archaellins, while crenarchaea can generally achieve motility with one filament protein. The archaella of the euryarchaeon *Haloferax volcanii* were shown to be made up of one major and one minor archaellin (Tripepi, Esquivel, Wirth, & Pohlschröder, 2013; Tripepi et al., 2010). A possibility is that some of the additional archaellins in euryarchaea are required as minor structural component, for example, to construct a hook complex at the base of the archaellum filament. The existence of this hook complex has been reported in one case, but no detailed studies on its composition or structure have been performed (Bardy, Mori, Komoriya, Aizawa, & Jarrell, 2002). Interestingly, a completely different explanation for the need of several archaellins came when studying the halophilic euryarchaeon *Haloarcula marismortui* (Syutkin, Pyatibratov, & Fedorov, 2014; Syutkin, Pyatibratov, Galzitskaya, Rodríguez‐Valera, & Fedorov, 2014).

**Figure 1:**
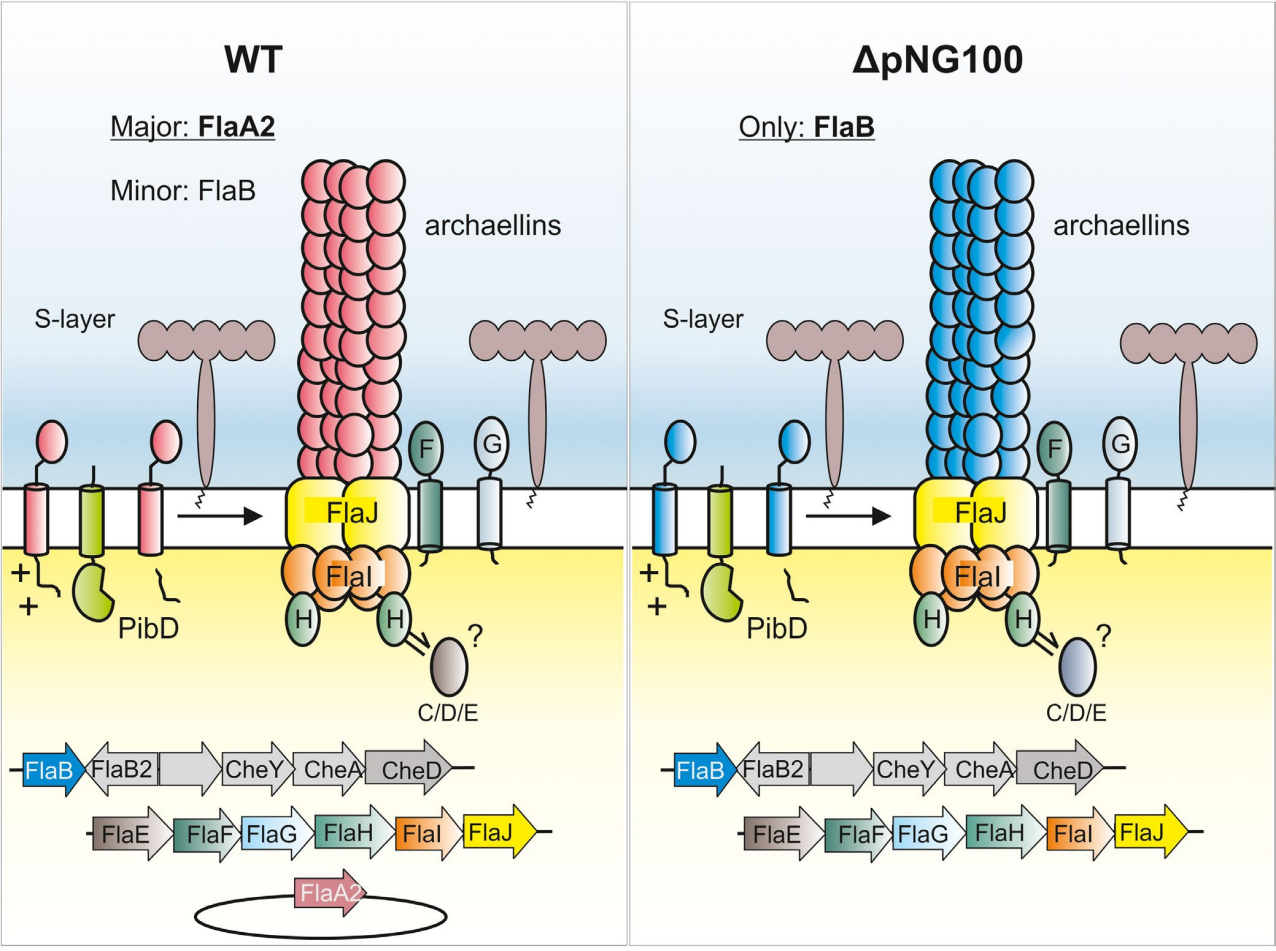
Schematic representation of archaella of the *Haloarcula marismortui* wild type and the ΔpNG100 strain. In the lower panel, the genomic locus of the archaellin genes is depicted. FlaB is encoded on the main chromosome next to the chemotaxis and assecory archaellin genes. FlaA2 is encoded on the plasmid pNG100. In wild‐type *H. marismortui*, the archaellum filament is majorly built of plasmid encoded FlaA2 archaellin, with minor amounts of FlaB. As *flaA2* is not present in the ΔpNG100 strain, the archaella filaments in this strain are exclusively made of FlaB. Archaellins are made as preproteins and N‐terminally cleaved by a PibD signal peptidase after which they are added at the base of the growing filament. Besides the archaellins, several archaellum assecory genes (*fla* genes) encode constituents of the archaellum motor (*flaHIJ*), the archaellum stator (*flaFG*) or have still an unknown function (*flaCDE*)

*Haloarcula marismortui* harbors four genes encoding archaellins on its genome. Two archaellins were found to constitute the archaellum filament; (a) *flaB* (WP_011224081.1) encoded on the major circular chromosome I, in the neighborhood of the *fla* accessory genes encoding components of the archaellum motor, and (b) *flaA2* (WP_011222137.1) on the pNG100 plasmid (~33 kbp) (see Figure 1) (Syutkin, Pyatibratov, Galzitskaya, et al., 2014). The function of the other two archaellin genes is unknown: *flaB2* (WP_049938970.1), located next to *flaB* on the main chromosome I, but transcribed from the opposite DNA strand, and *flaA1* (YP_137938.1), encoding a truncated archaellin without a leader peptide, located on chromosome II. The isolation of an *H. marismortui* strain that had lost the pNG100 plasmid (named ΔpNG100) enabled detailed characterizsation of the different archaellins (Pyatibratov et al., 2008). The wild type archaella are constituted mainly of the FlaA2 archaellin, encoded on the pNG100 plasmid, with minor amounts of the genomic encoded FlaB. In contrast, the archaella of the ΔpNG100 strain are built exclusively from the FlaB archaellin (Figure 1). It was demonstrated that both archaellins are able to form functional archaella alone (Syutkin, Pyatibratov, Beznosov, & Fedorov, 2012; Syutkin, Pyatibratov, Galzitskaya, et al., 2014). However, the FlaB protein proved very stable at high ionic strengths, while the FlaA2 protein can better resist media with low ionic strengths. Hence, it was proposed that these genes are ecoparalogs, that is, their products perform the same function under various environmental conditions (Syutkin, Pyatibratov, Galzitskaya, et al., 2014).

We have employed this interesting system to study regulation of motility. After varying the nutrient composition and salinity of the growth medium, we studied regulation of motility by studying archaellin transcription, translation, and correct assembly in to archaella. This study enabled us to identify the first environmental factors influencing archaellum expression in haloarchaea.

## 2 MATERIALS AND METHODS

### 2.1 Strains and growth conditions

*Haloarcula marismortui* B_1809 (ATCC 43049, DSM3752) was obtained from the All‐Russian Collection of Microorganisms (VKM), Pushchino. The culture was grown at 40°C on a shaking platform. The 30% salt medium consisted of 4 M NaCl, 0.6 M MgCl_2_*6H_2_O, 160 mM KCl, 10 mM CaCl_2_*2H_2_O, and 30 mM Bis‐Tris. For experiments with different salinities, the salt solutions with 20% and 25% salinities were prepared by dilution the 30% solution with pure water. For experiments with different carbon sources 1% yeast extract (rich media), 1% glucose or 1% tryptone were added to 25% salt solution. The pH of the media was adjusted to 7.0.

To study the influence of different carbon sources, cells of both strains were grown in rich medium (25% salinity, 1% yeast extract, 40°C) to mid‐log phase. Next the cells were pelleted, washed 3 times in the prewarmed final media and resuspended in media of 25% salinity containing either 1% yeast extract, 1% of glucose, or 1% of tryptone. Afterward, cells were incubated again at 40°C for 4 hr. Samples were taken at 4 hr after starvation.

### 2.2 qRT‐PCR

Total RNA was isolated from 2 ml culture taken at the indicated time‐points and conditions using the RNeasy Mini Kit (Qiagen, the Netherlands). The absence of genomic DNA was checked by PCR with primers (Hm_DNAcheck_fw and Hm_DNAcheck_rev). cDNA synthesis was performed using the First Strand cDNA synthesis Kit (Fermentas). Quantitative PCR analysis was carried out using an iTaq Universal SYBR green Supermix (Bio‐Rad) (real‐time PCR cycler Rotor‐GeneQ, Qiagen). Cq values (quantification cycle) for each transcript of interest were standardized to the Cq value of the housekeeping gene *gyrB*. Primer pairs used for qRT‐PCR reactions are listed in Table 1. At least two biological replicates and two technical replicates per reaction were performed. Relative quantification of gene expression was performed using the ΔΔ*C*_*t*_ method and a *t* test was performed to test if differences between different growth conditions were significant.

**Table 1:** Primers used in this study

Name	Sequence
Hm_DNAcheck_fw	GCGTGCTCCTGCTGTATTTC
Hm_DNAcheck_rev	GAGGAGCTGTAACACGATGG
Hm_flaA2_fw	GGGTGCATCTGATACCATTG
Hm_flaA2_rv	TTGGTCTCCACCATCTACTG
Hm_flaB_fw	CCAAGAAAGTAGCGACCAAG
Hm_flaB_rv	CTGACAGTTAGCGTTGATCC
Hm_flaI_fw	CGACGACTACGAGACGAATC
Hm_flaI_rv	GCTCGTCCTCATCAAGTTCC
Hm_gyrB_fw	ACATCGACGTAACGATTCAC
Hm_gyrB_rv	AGACCGGGTAGGACTTGTTG

### 2.3 Isolation of archaella

Archaella filaments were prepared by precipitation with polyethylene glycol 6,000 as described by Gerl, Deutzmann, and Sumper (1989). The protein preparations were dissolved in 50 mM Tris–HCl, pH 8.0, containing 20% NaCl. SDS‐PAGE (sodium dodecyl sulfate–polyacrylamide gel electrophoresis) was performed using 7% acrylamide gels. The proteins were stained with InstantBlue^TM^ (Expedeon).

### 2.4 Electron microscopy

Archaella samples were prepared for EM by negative staining with 1% uranyl acetate on Formvar‐ coated Copper grids. A grid was floated on a 20‐µl drop of archaella solution (about 0.01–0.05 mg/ml, in 3.4 M NaCl, 50 mM Tris–HCl, pH 8.0) for 2 min, blotted with filter paper, placed on top of a drop of 1% uranyl acetate and left for 1 min. Excess stain was removed by touching the grid to filter paper, and the grid was air dried. Samples were examined on a Jeol JEM‐1400 transmission electron microscope (JEOL, Japan) operated at 120 kV. Images were recorded digitally using a high‐resolution water‐cooled bottom‐mounted CCD camera.

### 2.5 Western blot analysis

Whole cell sample aliquots were harvested at exponential growth phase and equal amounts were loaded on 7% SDS‐PAGE and blotted on a polyvinylidene fluoride membrane (Thermo Fisher). Western blots with independent biological replicates were repeated at least three times. To detect archaellum components, polyclonal antibodies were raised in rabbit against FlaB archaellin protein. These antibodies were diluted 1:2,000 and the membranes incubated at 4°C overnight. After incubating the membranes with the secondary goat anti‐rabbit‐horseradish peroxidase antibody, Clarity Western ECL blotting substrate (Bio‐Rad) was added and chemiluminescent signals were recorded using an INTAS ECL Chemocam Imager (Intas, India).

#### 2.5.1 Chromatography mass spectrometry analysis

Proteins bands were excised and treated with Proteinase K (Promega) and trypsin (Sigma) at 37°C in a Thermo Mixer thermo shaker (Eppendorf, Germany). To stabilize proteinase K, CaCl_2_ was added to the solution to a final concentration of 5 mM. The molar ratio of enzyme‐to‐protein was 1/50. The reaction was stopped by adding trifluoroacetic acid to the solution. Prior to mass spectrometric analysis, the peptides were separated by reversed‐phase high‐performance liquid chromatography using an Easy‐nLC 1000 Nano liquid chromatography (ThermoFisher Scientific). The separation was carried out in a homemade column 25 cm in length and 100 μm in diameter packed with a C18 adsorbent; with an adsorbent particle size of 3 μm, and a pore size of 300 Å. The column was packed under laboratory conditions at a pressure of 500 atm. The peptides were eluted in a gradient of acetonitrile from 3% to 40% for 180 min; the mobile phase flow rate was 0.3 μl/min. Mass spectra of the samples were obtained using an OrbiTrap Elite mass spectrometer (Thermo Scientific, Germany). The peptides were ionized by electrospray at nano‐liter flow rates with 2 kV ion spray voltage; ion fragmentation was induced by collisions with an inert gas (collision induced dissociation in a high‐energy cell). The mass spectra were processed and peptides were identified using Thermo Xcalibur Qual Browser and PEAKS Studio (ver. 7.5) programs based on the sequences of UniRef‐100. Parent Mass Error Tolerance was 2.0 ppm and fragment Mass Error Tolerance was 0.1 Da. Only peptides were taken into account with a “10 l gP.” threshold value higher than 15.

## 3 RESULTS

**Figure 2:**
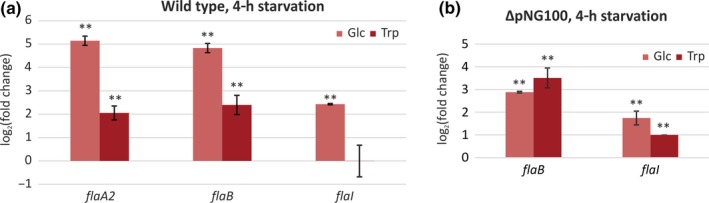
Transcription levels as measured by qRT‐PCR of tested genes, after growth for 4 h in the media with 1% glucose (Glc) or 1% of tryptone (Trp), in comparison with cells grown in media with 1% yeast extract. (a) Wild type. (b) ΔpNG100. Vertical bars show standard deviation between biological replicates. **Significantly different from growth in rich medium (*p* < 0.01)

We aimed at identifying differential regulation of archaellation in the wild type and the ΔpNG100 *H. marismortui* strain. Regulation of motility in archaea has been intensively studied in the crenarchaeon *S. acidocaldarius* (Albers & Jarrell, 2018). As starvation is an inducer of motility in *Sulfolobus*, first we aimed to analyze if growth on different carbon sources also induces archaellum expression in *H. marismortui*. We grew biological replicates of wild type and ΔpNG100 cells in liquid cultures at 40°C and transferred cells in exponential phase to fresh rich medium, or to medium containing glucose or tryptone as sole nutrient source. Cells were harvested 4 hr after changing the medium. qRT‐PCR was used to monitor mRNA levels of the *flaI* gene encoding the archaellum motor ATPase, in addition to the archaellin encoding *flaA2* and *flaB* genes, located on the pNG100 plasmid and the chromosome, respectively. mRNA levels of the genes of interest from cultures grown with a single carbon source were compared with those grown in rich medium (grown in the presence of yeast extract). In wild‐type *H. marismortui*, the expression of both *flaA2* and *flaB* archaellin genes was upregulated with a log_2_ fold of ~5 in glucose medium and a log_2_ fold of ~2 in tryptone medium when compared to medium with yeast extract (Figure 2a). *flaB* and *flaI* expression in the ΔpNG100 strain was slightly induced (log_2_ fold of ~3 and ~1.5 upregulation, respectively) both in medium with glucose or tryptone as sole nutrient source (Figure 2b). Conclusively, media containing different carbon sources resulted in a different expression of archaellin genes. However, the two strains responded similarly to changes of growth media (Figure S1a).

Next, we continued in search of environmental conditions that might influence motility and that would induce different responses in the two studied *H. marismortui* strains. As *H. marismortui* encounters varying salinity in its natural habitat, we hypothesized that stress induced by low ionic strength might influence motility and therefore archaellum regulation. To study the possible effect of different salinity on transcription of archaellum genes, we again used qRT‐PCR to evaluate mRNA levels. Biological replicates of the wt or ΔpNG100 strain were grown at 40°C in liquid cultures in 20%, 25%, and 30% salinity and cells were harvested at mid‐log phase. RNA was isolated and analyzed with qRT‐PCR. The transcription of the *flaI*,* flaA2* and *flaB* genes was monitored.

**Figure 3:**
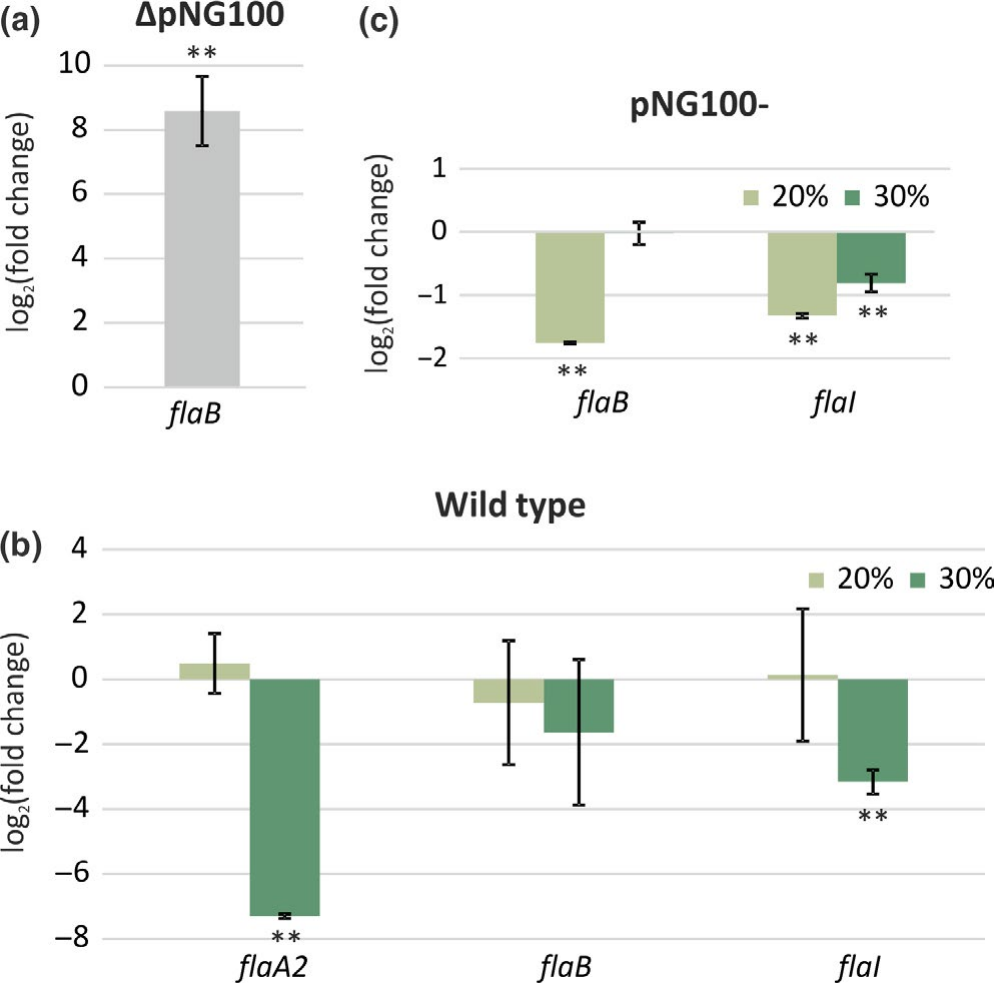
Transcription levels as measured by qRT‐PCR of tested genes media with different salinities. (a) Transcription level of the *flaB* gene in the ΔpNG100 strain, in comparison with the wt strain. (b) Change in the mRNA level in wild‐type cells grown in media with 20% and 30% salinity in comparison with those grown in media with 25% salinity. (c) Change in the mRNA level in ΔpNG100 cells grown in media with 20% and 30% salinity in comparison with those grown in media with 25% salinity. Vertical bars show standard deviation between biological replicates. **Significantly different from (a) wild type or (b) growth in medium with 25% salinity (*p* < 0.01)

First we compared the *flaB* level of the ΔpNG100 strain with that of the wild type in the same medium containing 25% salinity (standard growth conditions). *flaB* transcription could be detected in both strains, but the *flaB* levels were upregulated with a log_2_ fold of ~8 in the ΔpNG100 strain compared to the wild type, indicating that *flaB* expression is strongly repressed in the presence of pNG100 (Figure 3a).

Next, we compared transcription of *flaB*,* flaA2* and *flaI* in medium with 20% and 30% salinity, to that of the standard medium with 25% salinity. Previous work has shown that the archaella of the wild‐type strain grown under optimal conditions are mainly formed by FlaA2 (Syutkin, Pyatibratov, & Fedorov, 2014). In the wild type, expression of *flaA2* was strongly decreased at 30% salinity compared with those at 25%. In addition expression of *flaI* was also found to be downregulated (Figure 3b), whereas *flaB* transcription was not affected. In medium with 20% salinity, the expression of archaellum genes was similar to that in medium with 25% salinity (Figure 3b). The ΔpNG100 strain lacks the *flaA2* gene and therefore only expression of *flaB* and *flaI* was studied. In contrast to the wild‐type strain, mRNA levels of both genes were slightly lower in the medium with 20% salinity compared to that of 25% (Figure 3c). Increasing the ionic strength to 30% salinity did not alter the expression of archaellum genes. Thus, in the wild‐type archaellin *flaA2* expression is repressed at high ionic strength. Comparison between the ΔpNG100 strain and the wild type shows that expression of *flaB* and *flaI* increases at higher ioning strength specifically in the ΔpNG100 strain (Figure S1b).

**Figure 4:**
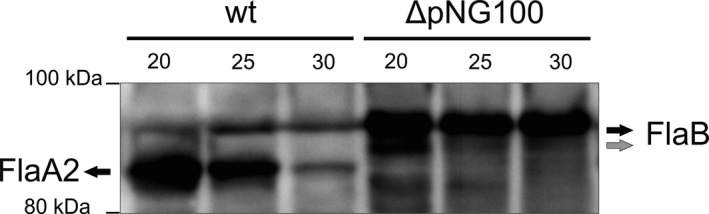
Immuno‐blot detecting the presence of archaellins FlaB and FlaA2 in cellular lysates of wild type and ΔpNG100 strains at different salinities. Numbers above the lanes indicate the % salinity of the growth medium. Gray arrow, FlaB protein with a possibly different glycosylation pattern (see text)

As clear differences in archaellin transcription between the two strains were observed under growth in medium with different salinities, in a next step we aimed to study the translation of archaellins. SDS‐PAGE and Western blot analysis was performed on total cell lysate of the same cultures as used for the qRT‐PCR experiments. Antibodies recognizing both the FlaA2 and FlaB protein were used for immunodetection (see M&M). FlaA2 and FlaB can be distinguished by their size on SDS polyacrylamide gels: FlaA, 47 kDa and FlaB, 48 kDa, (the archaellins of *H. marismortui* run differently on SDS‐gel because of their low pI and protein modifications, Pyatibratov et al., 2008; Syutkin, Pyatibratov, Galzitskaya, et al., 2014). In wild‐type cultures, we detected minor amounts of FlaB and higher levels of FlaA2, which is in correspondence with the qRT‐PCR results (Figures 3a and 4). While the low levels of FlaB seemed constant under different salinities, FlaA2 levels varied significantly. At 20% salinity, the FlaA2 signal was much increased compared to 25%, while at 30% salinity, the FlaA2 signal was reduced (Figure 4). This decrease in FlaA2 protein levels at 30% salinity is corresponding with the decreasing mRNA levels as measured by qRT‐PCR under these conditions. At 20% salinity, the increase in FlaA2 protein compared with 25% salinity was only measured with Western blot and was not observed at the mRNA level via qRT‐PCR.

After the wild type, we also analyzed the ΔpNG100 strain that lacks *flaA2* with Western blot. The FlaB signal was in all tested conditions much more intense as the FlaB signal of the wild type, corresponding with the qRT‐PCR levels (Figures 2a and 3). At 20% salinity, a small band was detected just below the FlaB band, which might represent a not completely glycosylated form or proteolysis products of intracellular FlaB (Figure 4). The intensity of the FlaB signal was approximately similar at 20%, 25%, and 30% salinity, corresponding with the qRT‐PCR results, which indicated no strong regulation at different salinities in the ΔpNG100 strain.

**Figure 5:**
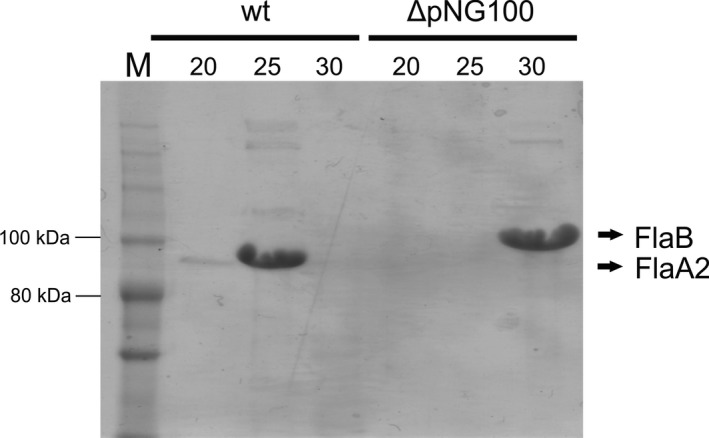
SDS‐PAGE of isolated archaella from wild type and ΔpNG100 strains at different salinities. Numbers above the lanes indicate the % of salinity of the growth medium. M, marker

**Figure 6:**
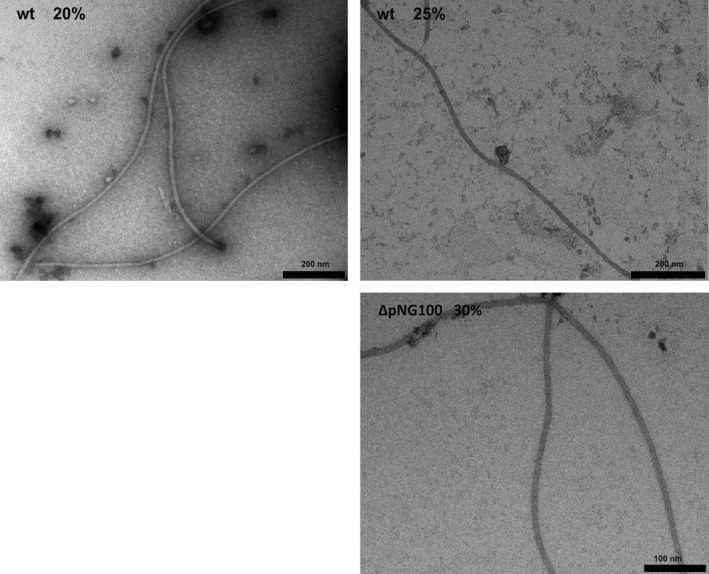
Electron micrographs of isolated archaella fraction of the *Haloarcula marismortui* wild type (wt) or the ΔpNG100 strains at different salinities at which archaella were produced (20% and 25% for wt and 30% for ΔpNG100). Error bars upper panel, 200 nm and lower panel, 100 nm

After measuring transcription and translation of archaellin genes in conditions with different ionic strengths, we proceeded to analyze their correct assembly of the proteins in archaella represented on the cell surface. To this end, extracellular archaella were isolated from both *H. marismortui* strains grown to stationary phase at 40°C in liquid media with different salinities. The samples were analyzed by SDS‐PAGE on which again FlaA2 and FlaB could be distinguished based on their electrophoretic mobility (Figure 5). The identity of the proteins was verified with Mass Spectrometry analysis (see Materials and Methods). This confirmed (>90% coverage, 340 unique peptides) that the major protein bands in the wild‐type samples at 20% and 25% salinity represented archaellin A2: “*H. marismortui* Flagellin protein A2 (Q5V881_HALMA)” (Figure S2). The integrity of archaella was checked by Electron Microscopy (Figure 6). The wild‐type strain produced most archaella in its regular growth medium with 25% salinity, which is demonstrated by the intense FlaA2 band on the SDS gel. In medium with 20% salinity, a low amount of archaella was detected and no archaella could be isolated from cells growing at 30% salinity. The archaella of the ΔpNG100 strain were formed exclusively of FlaB. The archaella produced by ΔpNG100 were only detected in medium with 30% salinity. In addition, using optical microscopy we confirmed that only in the media where archaella could be isolated, cells were motile (data not shown). The strong differences between the amount of successfully assembled and secreted archaellins is not reflected in the intracellular archaellin levels as measured by qRT‐PCR and Western blot. This suggests that an additional step of regulation might occur at the level of assembly of the filament.

## 4 DISCUSSION

Archaea achieve motility with help of a specialized motility structure, the archaellum. In recent years significant progress has been made revealing the structural details of the archaellum. In contrast, regulation of archaellation has only been studied in a handful of model organisms. With this work, we expand the knowledge on archaeal regulation of motility using the halophilic archaeon *H. marismortui*. We show that growth of *H. marismortui* in media with different carbon sources influences the expression of archaellum genes. Moreover, we identified salinity as environmental factor inducing archaellation. By studying transcription, translation, and assembly of archaellins, regulation of archaellation was studied at different levels. Besides the wild‐type strain, this study also included the ΔpNG100 strain, to allow for the better characterization of regulation of the two ecoparalogous archaellins: FlaB, encoded on the main chromosome, and FlaA2, encoded on the plasmid pNG100.

It was shown that transcription and translation of the *flaA2* gene in the wild type are optimal at low salinity and are reduced at higher salinities. In contrast, the ΔpNG100 strain showed under the same conditions only subtle or no differences of *flaB* mRNA and intracellular FlaB archaellin concentration. The highest number of archaella in the wild type could be isolated at 25% salinity and in the ΔpNG100 strain at 30% salinity. Interestingly, FlaB (pre)‐archaellins could be detected by immunoblotting in total cell lysates of cultures grown at all three salinities. This indicates that the archaellins are produced at different ionic strengths, but that their assembly in correct archaella occurs mainly in medium of a specific salinity. Thus, even though archaellins seem to be produced intracellularly, their secretion and assembly in archaella is subject to an additional level of regulation.

The differential regulation of archaellation in the wild type and ΔpNG100 strain fit very well to the suggested role of their ecoparalogous archaellins. FlaB was previously shown to be more stable at high ionic strength (Pyatibratov et al., 2008). Indeed in the ΔpNG100 strain, where archaella are constituted exclusively of FlaB, the yield of archaella directly correlates with increased ionic strength. During a previous study, it remained unclear why the wild‐type strain possessed archaella built almost exclusively of FlaA2. In this present work, we could show that this is the result of strong transcriptional inhibition of *flaB* in wild‐type *H. marismortui*. The higher stability at high ionic strengths of archaella built exclusively of FlaB raises the question why in the wild‐type strain pNG100 encoded FlaA2 has substituted FlaB as major archaellin and what is the evolutionary advantage of maintaining FlaA2. It was shown previously that the archaellin FlaA2 is more stable at low ionic strengths (Syutkin, Pyatibratov, Galzitskaya, et al., 2014) and indeed the above described analysis shows that its expression is induced in media with 20% salinity. As especially low ionic strength causes stress to halophilic organisms, the presence of the FlaA2 archaellin, and specifically the possibility to adapt the occurrence of FlaB and FlaA2 in archaella, represents a distinct advantage.

The observation of the two ecoparalogous archaellins in *H. marismortui* resembles that of *Haloferax volcanii*, which encodes six different pilins. It was shown that all six pilins could individually build pili, but that these pili had slightly different functionalities (Esquivel, Xu, & Pohlschröder, 2013). While some pili attached even better to surfaces as the wild type pili, others could not adhere anymore (Esquivel et al., 2013; Pohlschröder & Esquivel, 2015). These findings, in combination with the present study, indicate that, at least in haloarchaea, multiple encoded pilins and archaellins are not redundant, but in fact have subtle functional differences that allow cells to cope with varying environmental conditions.

## CONFLICT OF INTEREST

The authors declare no conflict of interest.

## AUTHOR CONTRIBUTION

AS, MP, TEFQ, S‐VA designed research. ASS, AKS, and MP performed experiments. MW analyzed qRT‐PCR data. All authors interpreted data and contributed to writing the article.

## Data Availability

The datasets generated and analyzed during this study are available from the corresponding authors T.E.F.Q and A.S.S. on reasonable request.
