# Supplementary material for: Salt‐dependent regulation of archaellins in Haloarcula marismortui
Source: Microbiologyopen. 2018 Oct 1;8(5):e00718. doi: 10.1002/mbo3.718 (PMC6528647; doi:10.1002/mbo3.718)
Supplement: Supplementary file 1 [file MBO3-8-e00718-s001.docx]

**Supplementary material**


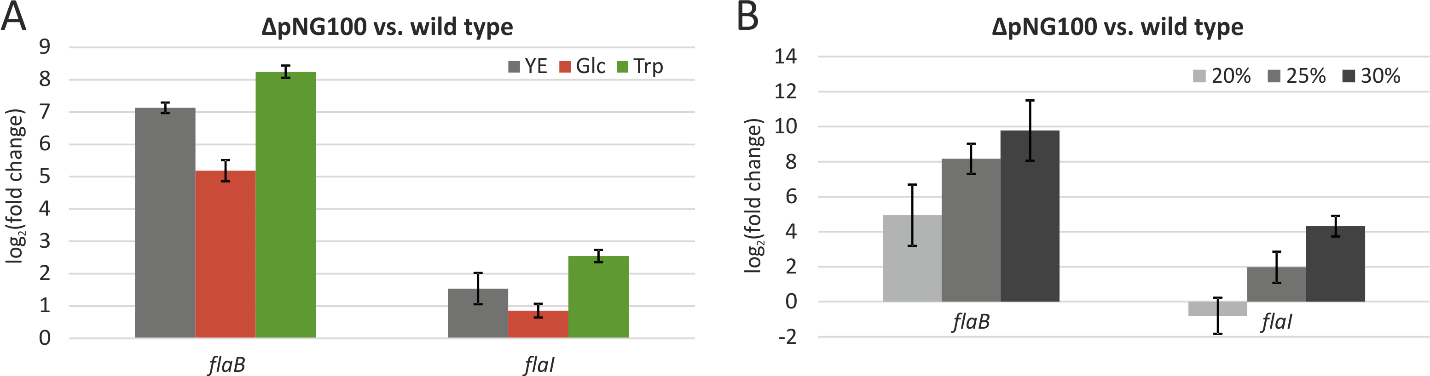


Fig S1 Comparison of transcription levels in the wild type and ΔpNG100 strain as measured by qRT-PCR of tested genes. A) Transcription level of the *flaB* and *flaI* gene in the ΔpNG100 strain, in comparison with the wt strain after growth for 4 h in the media with 1 % glucose (Glc), 1 % of tryptone (Trp), or 1 % yeast extract. B) Transcription level of the *flaB* and *flaI* gene in the ΔpNG100 strain, in comparison with the wt strain after growth in media with different salinity.


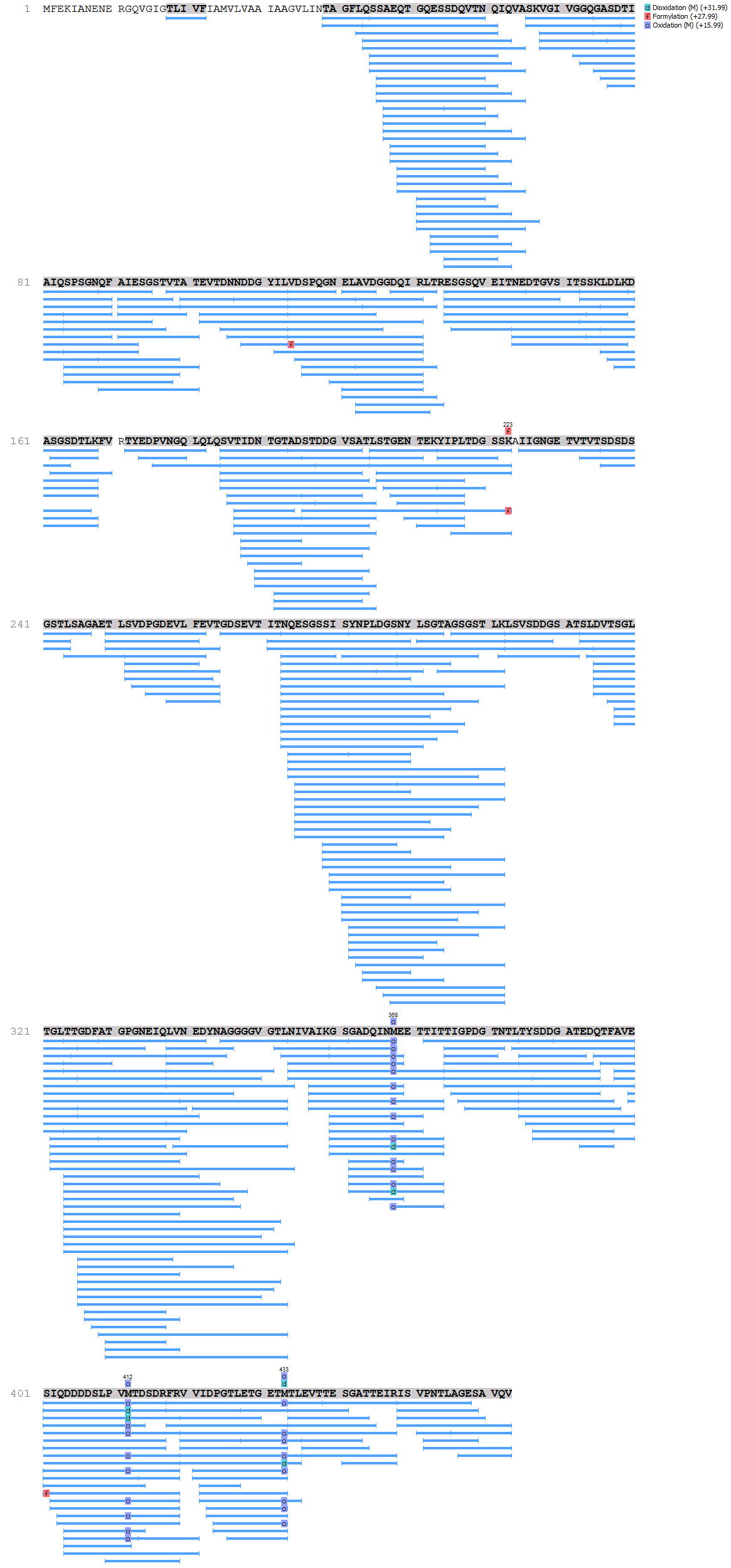
Figure S2

Figure S2. Mass Spectrometry analysis of most prominent protein in archaella isolated from wild type *Haloarcula marismortui* in medium with 20 % and 25% salinity. Protein coverage of Flagellin A protein of *Haloarcula marismortui* (Q5V881_HALMA) is shown. In gray the full protein sequence is shown. In blue the unique peptides are depicted.
